# Environmental Data Do Not Correlate With Plant Genetic Diversity in Alpine Ecosystems

**DOI:** 10.1002/ece3.71332

**Published:** 2025-05-07

**Authors:** J. L. Blanco‐Pastor, J. Fajardo, A. G. Fernández de Castro, M. Fernández‐Mazuecos, J. M. García‐Martín, I. M. Liberal, A. Otero, J. V. Sandoval‐Sierra, I. Villa‐Machío, J. C. Zamora, S. Martín‐Bravo

**Affiliations:** ^1^ Departamento de Biología IVAGRO, Universidad de Cádiz, Campus de Excelencia Internacional Agroalimentario (ceiA3) Cádiz Spain; ^2^ Department of Biodiversity and Conservation Real Jardín Botánico‐CSIC Madrid Spain; ^3^ Department of Animal Biology, Ecology, Parasitology, Edaphology and Agricultural Chemistry University of Salamanca Salamanca Spain; ^4^ Departamento de Biología, Facultad de Ciencias Universidad Autónoma de Madrid Madrid Spain; ^5^ Conservatoire et Jardin botaniques de la Ville de Genève Pregny‐Chambésy Switzerland; ^6^ Department of Molecular Biology and Biochemical Engineering Universidad Pablo de Olavide Seville Spain

**Keywords:** Alps, climate change, conservation, environmental data, genetic diversity, high mountains, plant biodiversity

## Abstract

Genetic diversity is a fundamental asset for populations to adapt to changing environmental conditions, particularly under climate change. Although much attention has been paid to protecting taxonomic and ecological diversity, genetic diversity has often been overlooked in management and conservation plans due to the difficulty and costs of its evaluation. We expect an extraordinary impact of global warming on alpine habitats and species that urges us to prioritize the protection of genetic diversity. We analyzed the relationship between 48 environmental factors (climate, soil, and topography) and genetic diversity with AFLP data from 309 populations of 14 European alpine plant species. We used LASSO models and univariate linear regressions to investigate associations between genetic diversity and rarity values in populations as a function of environmental factors at sampling sites, and to identify the best environmental predictors. We found that among all factors, only a topographic descriptor associated with surface concavity and convexity (profile curvature, pcurv) had minimal but significant effects on heterozygosity and genetic rarity when combining all populations from all species (*r* ^2^ = 0.022 and *r* ^2^ = 0.017, respectively). When we analyzed the species independently, only *Saponaria pumila* (Caryophyllaceae) and *Androsace vitaliana* (Primulaceae) showed significant and marginally significant associations between heterozygosity and pcurv (*p*‐value = 0.036, *r* ^2^ = 0.126 and *p*‐value = 0.086, *r* ^2^ = 0.093, respectively). Further analyses pointed to a shared spatial autocorrelation between heterozygosity and pcurv in these species. No significant associations were observed between pcurv and the genetic diversity indexes of the remaining species analyzed. We found that, in general terms, the environment in European alpine areas does not drive the distribution of genetic diversity of plant species. We stress the need for species‐specific data and detailed assessments of selectively neutral and adaptive genetic markers to inform conservation efforts to meet global biodiversity protection targets for 2030 in high mountain ecosystems.

## Graphical abstract

This study investigates the impact of environmental factors on genetic diversity in 14 European alpine plant species using AFLP data. Among 48 environmental factors, only surface curvature (pcurv) showed a minimal but significant effect on heterozygosity and genetic rarity across species, with stronger associations observed in *S. pumila* and *A. vitaliana*, likely due to spatial autocorrelations. No significant associations were found for other species.

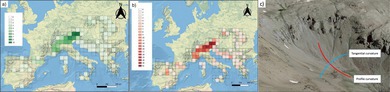

## 1 Introduction

High mountain ecosystems provide key ecosystem services and high levels of taxonomic biodiversity that are particularly sensitive to anthropogenic climate change (Parmesan 2006; Pauli et al. 2012). Studies involving the effect of global warming on high mountain species have detected a general pattern of range contraction, as species move upward in elevation (Pauli et al. 1996; Walther et al. 2005; Thuiller et al. 2005; Engler et al. 2011; Gottfried et al. 2012). Consequently, under the current global warming scenario, the extension of alpine habitats could decline drastically and lead to multiple species extinctions (Parmesan 2006). Since the first decade of the twentieth century, alpine plants have become rarer on the European continent, and lower‐altitude plants have become more frequent at higher altitudes in response to global warming (Pauli et al. 2012). This is the first stage of what has been called the “taxonomic homogenization” process (McKinney and Lockwood 1999; Olden and Rooney 2006). In this context, conservation studies have revealed that alpine ecosystems are a priority, but it is still unclear which particular alpine areas deserve the most attention.

Genetic diversity can influence the ability of populations to adapt to changing environmental conditions. Although much attention has been paid to the protection of taxonomic (especially at the species level) and ecological (i.e., habitat, ecosystem) diversity, little attention has been paid to the protection of genetic diversity. Many management and conservation plans have not considered this dimension of biodiversity (Laikre 2010; Laikre et al. 2020; Hoban et al. 2021), even though it is included among the main goals (goal A, “Protect and Restore”) and targets (target 4, “Halt Species Extinction, Protect Genetic Diversity, and Manage Human‐Wildlife Conflicts”) of the Kunming‐Montreal Global Biodiversity Framework (https://www.cbd.int/gbf/). Previous research has shown that, for particular alpine plant species, the geographic distribution of genetic diversity can be associated with environmental factors, such as climate, soil, and topography (Hughes et al. 2008; Alvarez et al. 2009; Crawford and Whitney 2010; Manel et al. 2012; Graae et al. 2018; Blanco‐Pastor et al. 2019). The impact of these environmental factors on the distribution of plant genetic diversity has been explored for particular species at the local scale (Yang et al. 2012; Li et al. 2012; Kutnjak et al. 2014; Sanz et al. 2014; Luo et al. 2016; Wasowicz et al. 2016) but to date, it is not known to what extent we can identify medium‐ to large‐scale patterns of genetic diversity across species from environmental variables, or if we could use them for decision‐making to maximize genetic diversity protection in conservation actions. The conservation of populations with high genetic diversity is considered a priority in programs aiming at the long‐term viability of the species (Kukkala and Moilanen 2013). However, measuring cross‐species genetic diversity at medium or large scales can be challenging, given that detecting intraspecific genetic differences often requires dedicated funding for advanced genetic tools and extensive sampling (Kardos et al. 2021). As a result, prioritizing which populations to protect becomes a complex decision‐making process.

Environmental data are often more readily available and accessible than genetic data, making it a practical option for predicting areas with high genetic diversity. Here we hypothesize that data from population sites (climate, topography, or soil) could be used to identify populations with high genetic diversity in alpine environments. In this study, we analyzed genetic data (amplified fragment length polymorphisms, AFLPs) of 309 populations from 14 European alpine plant species, only occurring above the tree line, to model relationships between environmental factors (soil, climate, and topography) at sampling sites and genetic diversity and rarity of the populations. We aim to use these data to assess the conservation priorities of European alpine plant species, thereby guiding decision‐making and conservation efforts.

## 2 Methods

### 2.1 Genetic Data

**FIGURE 1:**
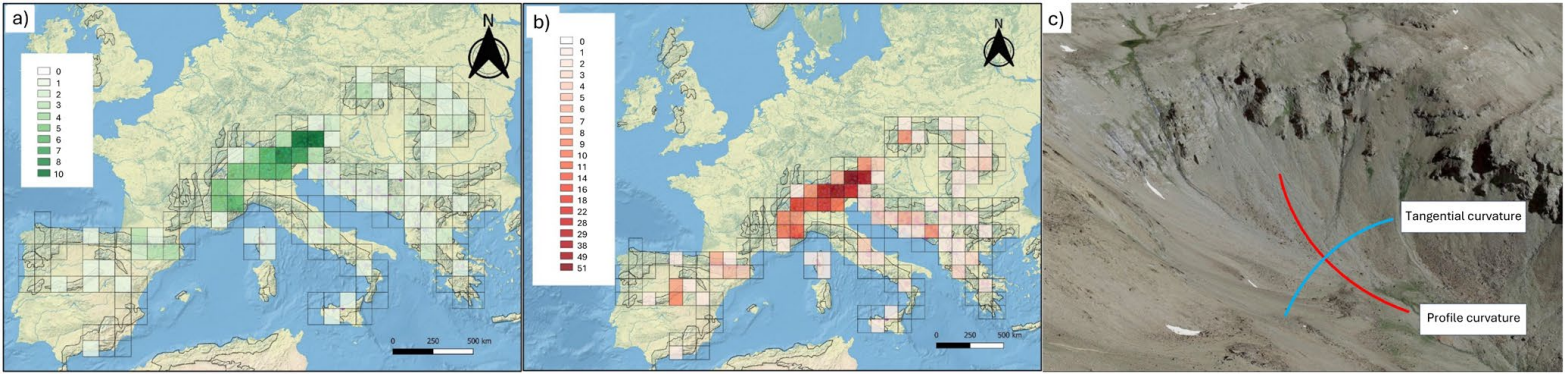
Map of the European area studied, representing the number of species (a) and populations (b) per cell used in our study; (c) profile and tangential curvatures (pcurv and tcurv) identify concavity and convexity in the direction of the slope, or perpendicular to the slope (see Amatulli et al. 2018). The profile curvature measures the change rate of slope along a flow line. It affects the acceleration or deceleration of water flow along the surface and thus influences erosion and deposition of soil. The tangential curvature measures the change rate perpendicular to the slope gradient and is related to the convergence and divergence of flow across a surface (Stefano et al. 2000; Wilson and Gallant 2000). The units of both curvatures are radians per meter, where positive and negative values indicate convex and concave surfaces, respectively. Concave curvature promotes soil deposition while convex curvature promotes soil erosion.

We obtained genetic information (AFLPs) of 309 populations from 14 alpine angiosperm species from Europe (Figure 1a,b), representing 6 families (Primulaceae, Plumbaginaceae, Asteraceae, Brasssicaceae, Caryophyllaceae, Campanulaceae). Datasets of these species were obtained after a literature search in the *Scopus* database (https://www.scopus.com/) with the terms: *TITLE‐ABS‐KEY (((alpine OR mountain*) *AND (genetic OR phylogeography) AND NOT (virus OR bacteria OR cultivar OR landrace OR livestock))) AND SUBJAREA (mult OR agri OR bioc OR immu OR neur OR phar*). We selected species from the European alpine region with a distribution range above the timberline and with precise occurrence data of the sampled populations available. We only considered datasets with a sample size of ≥ 5 populations and ≥ 5 individuals per population.

The AFLP procedure in all filtered studies began by extracting genomic DNA and digesting it with the restriction enzymes EcoRI and MseI, followed by ligation to double‐strand adapters with T4 DNA ligase. The restriction‐ligation reaction was incubated, typically at 37°C for 2 to 3 h, and then a preselective amplification was performed with adapters and primers according to the AFLP Ligation and Preselective Amplification Module protocol (PE Applied Biosystems, Foster City, California, USA). This was followed by selective amplification with primer combinations labeled with fluorescent dyes for detection. Initial screenings of several primer combinations were conducted, and those that yielded clear, reproducible bands were selected for further analysis. The amplified products were separated by polyacrylamide gel electrophoresis on automated sequencers, with size calibration performed with an internal size standard (GeneScan500 [ROX], PE Applied Biosystems). The AFLP fragments were then scored with Genographer 1.6.0, with ambiguous peaks excluded and low‐intensity but clear peaks included in the analysis. The results were exported as a presence/absence matrix, and reproducibility tests, including replicates and negative controls, were used to ensure data accuracy. The main differences among species involved slight variations in reaction volumes, primer combinations, and electrophoresis techniques. The AFLP data matrices ranged from 161 to 659 loci and from 5 (min) to 56 (max) populations (see the number of populations and loci for each species in Appendix S1 and the AFLP data matrices in Appendix S2).

### 2.2 Analysis of Population Genetic Diversity and Rarity

We used Nei's measure of the average gene diversity per locus *H*_*S*_ (Nei et al. 1973) with a correction for small samples (Kosman 2003) to analyze heterozygosity in our 309 study populations separately. We also estimated per‐population genetic rarity as the inverse of the number of populations in which each allele occurs averaged across alleles for each target population (Taberlet et al. 2012). High genetic rarity would indicate the presence of alleles in a limited number of populations.

### 2.3 Environmental Variables

We obtained information for our 309 study populations on 48 climatic, topographic, and edaphic factors at 1‐km resolution from the following public databases: CHELSA (https://chelsa‐climate.org/downloads/), EARTHENV (https://www.earthenv.org/topography), and ISRIC (https://files.isric.org/soilgrids/latest/data_aggregated/1000m/) (see Table 1). The environmental layers were cropped to the extent of our population datasets. Then we created a collection of layers with the stack() function of the R package *raster* (Hijmans 2018) and extracted the environmental values at population sites from their GPS coordinates (see environmental data matrices in Appendix S3).

**TABLE 1:** Information on the environmental variables used in this study, including their names, descriptions, and sources.

Variable	Description	Source	References
aspectcosine_1KMmn_GMTEDmd	Aspect Cosine	https://www.earthenv.org/topography	Amatulli et al. (2018)
aspectsine_1KMmn_GMTEDmd	Aspect Sine	https://www.earthenv.org/topography	Amatulli et al. (2018)
bdod_0.5cm_mean_1000	Bulk density of the fine earth fraction	https://files.isric.org/soilgrids/latest/data_aggregated/1000m/	Batjes et al. (2020)
cec_0.5cm_mean_1000	Cation Exchange Capacity of the soil	https://files.isric.org/soilgrids/latest/data_aggregated/1000m/	Batjes et al. (2020)
cfvo_0.5cm_mean_1000	Volumetric fraction of coarse fragments (> 2 mm)	https://files.isric.org/soilgrids/latest/data_aggregated/1000m/	Batjes et al. (2020)
CHELSA_bio1_1981.2010_V.2.1	Mean annual daily mean air temperatures averaged over 1 year	https://chelsa‐climate.org/downloads/	Karger et al. (2017)
CHELSA_bio10_1981.2010_V.2.1	The warmest quarter of the year is determined (to the nearest month)	https://chelsa‐climate.org/downloads/	Karger et al. (2017)
CHELSA_bio11_1981.2010_V.2.1	The coldest quarter of the year is determined (to the nearest month)	https://chelsa‐climate.org/downloads/	Karger et al. (2017)
CHELSA_bio12_1981.2010_V.2.1	Accumulated precipitation amount over 1 year	https://chelsa‐climate.org/downloads/	Karger et al. (2017)
CHELSA_bio13_1981.2010_V.2.1	The precipitation of the wettest month.	https://chelsa‐climate.org/downloads/	Karger et al. (2017)
CHELSA_bio14_1981.2010_V.2.1	The precipitation of the driest month.	https://chelsa‐climate.org/downloads/	Karger et al. (2017)
CHELSA_bio15_1981.2010_V.2.1	The Coefficient of Variation is the standard deviation of the monthly precipitation estimates expressed as a percentage of the mean of those estimates (i.e., the annual mean)	https://chelsa‐climate.org/downloads/	Karger et al. (2017)
CHELSA_bio16_1981.2010_V.2.1	The wettest quarter of the year is determined (to the nearest month)	https://chelsa‐climate.org/downloads/	Karger et al. (2017)
CHELSA_bio17_1981.2010_V.2.1	The driest quarter of the year is determined (to the nearest month)	https://chelsa‐climate.org/downloads/	Karger et al. (2017)
CHELSA_bio18_1981.2010_V.2.1	The warmest quarter of the year is determined (to the nearest month)	https://chelsa‐climate.org/downloads/	Karger et al. (2017)
CHELSA_bio19_1981.2010_V.2.1	The coldest quarter of the year is determined (to the nearest month)	https://chelsa‐climate.org/downloads/	Karger et al. (2017)
CHELSA_bio2_1981.2010_V.2.1	Mean diurnal range of temperatures averaged over 1 year	https://chelsa‐climate.org/downloads/	Karger et al. (2017)
CHELSA_bio3_1981.2010_V.2.1	Ratio of diurnal variation to annual variation in temperatures	https://chelsa‐climate.org/downloads/	Karger et al. (2017)
CHELSA_bio4_1981.2010_V.2.1	Standard deviation of the monthly mean temperatures	https://chelsa‐climate.org/downloads/	Karger et al. (2017)
CHELSA_bio5_1981.2010_V.2.1	The highest temperature of any monthly daily mean maximum temperature	https://chelsa‐climate.org/downloads/	Karger et al. (2017)
CHELSA_bio6_1981.2010_V.2.1	The lowest temperature of any monthly daily mean maximum temperature	https://chelsa‐climate.org/downloads/	Karger et al. (2017)
CHELSA_bio7_1981.2010_V.2.1	The difference between the Maximum Temperature of Warmest month and the Minimum Temperature of Coldest month	https://chelsa‐climate.org/downloads/	Karger et al. (2017)
CHELSA_bio8_1981.2010_V.2.1	The wettest quarter of the year is determined (to the nearest month)	https://chelsa‐climate.org/downloads/	Karger et al. (2017)
CHELSA_bio9_1981.2010_V.2.1	The driest quarter of the year is determined (to the nearest month)	https://chelsa‐climate.org/downloads/	Karger et al. (2017)
clay_0.5cm_mean_1000	Proportion of clay particles (< 0.002 mm) in the fine earth fraction	https://files.isric.org/soilgrids/latest/data_aggregated/1000m/	Batjes et al. (2020)
dx_1KMmn_GMTEDmd	First order partial derivative (E–W slope)	https://www.earthenv.org/topography	Amatulli et al. (2018)
dxx_1KMmn_GMTEDmd	Second order partial derivative (E–W slope)	https://www.earthenv.org/topography	Amatulli et al. (2018)
dy_1KMmn_GMTEDmd	First order partial derivative (N–S slope)	https://www.earthenv.org/topography	Amatulli et al. (2018)
dyy_1KMmn_GMTEDmd	Second order partial derivative (N–S slope)	https://www.earthenv.org/topography	Amatulli et al. (2018)
eastness_1KMmn_GMTEDmd	Eastness	https://www.earthenv.org/topography	Amatulli et al. (2018)
elevation_1KMmn_GMTEDmn	Elevation	https://www.earthenv.org/topography	Amatulli et al. (2018)
nitrogen_0.5cm_mean_1000	Total nitrogen (N)	https://files.isric.org/soilgrids/latest/data_aggregated/1000m/	Batjes et al. (2020)
northness_1KMmn_GMTEDmd	Northness	https://www.earthenv.org/topography	Amatulli et al. (2018)
ocd_0.5cm_mean_1000	Organic carbon density	https://files.isric.org/soilgrids/latest/data_aggregated/1000m/	Batjes et al. (2020)
ocs_0.30cm_mean_1000	Organic carbon stocks	https://files.isric.org/soilgrids/latest/data_aggregated/1000m/	Batjes et al. (2020)
pcurv_1KMmn_GMTEDmd	Profile curvature	https://www.earthenv.org/topography	Amatulli et al. (2018)
roughness_1KMmn_GMTEDmd	Roughness	https://www.earthenv.org/topography	Amatulli et al. (2018)
sand_0.5cm_mean_1000	Proportion of sand particles (> 0.05 mm) in the fine earth fraction	https://files.isric.org/soilgrids/latest/data_aggregated/1000m/	Batjes et al. (2020)
silt_0.5cm_mean_1000	Proportion of silt particles (≥ 0.002 mm and ≤ 0.05 mm) in the fine earth fraction	https://files.isric.org/soilgrids/latest/data_aggregated/1000m/	Batjes et al. (2020)
slope_1KMmn_GMTEDmd	Slope	https://www.earthenv.org/topography	Amatulli et al. (2018)
soc_0.5cm_mean_1000	Soil organic carbon content in the fine earth fraction	https://files.isric.org/soilgrids/latest/data_aggregated/1000m/	Batjes et al. (2020)
tcurv_1KMmn_GMTEDmd	Tangential curvature	https://www.earthenv.org/topography	Amatulli et al. (2018)
tpi_1KMmn_GMTEDmd	Topographic Position Index	https://www.earthenv.org/topography	Amatulli et al. (2018)
tri_1KMmn_GMTEDmd	Terrain Ruggedness Index	https://www.earthenv.org/topography	Amatulli et al. (2018)
vrm_1KMmn_GMTEDmd	Vector Ruggedness Measure	https://www.earthenv.org/topography	Amatulli et al. (2018)
wv0010_0.5cm_mean_1000	Water retention volumetric—10 kPa	https://files.isric.org/soilgrids/latest/data_aggregated/1000m/	Batjes et al. (2020)
wv0033_0.5cm_mean_1000	Water retention volumetric—33 kPa	https://files.isric.org/soilgrids/latest/data_aggregated/1000m/	Batjes et al. (2020)
wv1500_0.5cm_mean_1000	Water retention volumetric—1500 kPa	https://files.isric.org/soilgrids/latest/data_aggregated/1000m/	Batjes et al. (2020)

### 2.4 Correlations, LASSO Models and Univariate Linear Regressions

We performed pairwise correlations among all genetic and environmental variables. To investigate whether a proportion of variance in genetic diversity and rarity across our study populations could be predicted by a regularized subset of environmental factors, and to identify the best predictor variables, we fit a Gaussian generalized linear model (GLM) via penalized maximum likelihood with LASSO penalty (Tibshirani 1996). LASSO is a regularization technique used in regression analysis that helps in variable selection and model simplification. It shrinks the coefficient estimates toward zero as the regularization parameter lambda (*λ*) increases, allowing efficient identification of the best model that involves the smallest subset of parameters. We used a 1000th‐fold cross‐validation to identify the best value for the lambda parameter. We performed the GLM fit and cross‐validation with the R package *glmnet* (Friedman et al. 2010; Simon et al. 2011).

To understand the individual relationships between the best environmental variable selected by LASSO and genetic diversity (heterozygosity and rarity), and to assess the practical use of our approach to identify high cross‐species diversity hotspots from single environmental variables, we regressed the best LASSO variable against the heterozygosity and rarity of all populations from all species pooled. Then, to investigate associations at the species level, we ran these linear models independently. We selected six species with more than 20 populations to gain statistical power and precision and reduce Type I and Type II errors.

To identify the impact of spatial autocorrelation in our results, we calculated the Moran's I index of heterozygosity and pcurv for each species in the dataset with the R package *spdep* (Bivand and Wong 2018; Bivand 2022). Spatial weights were generated from k‐nearest neighbors (*k* = 4), and Moran's I was calculated for each variable from these spatial weights. The analysis was performed separately for each species, and results were then visualized in bar plots, where Moran's I values for each species and variable were compared.

## 3 Results

### 3.1 Associations Between Genetic Diversity and the Multivariate Environment

**FIGURE 2:**
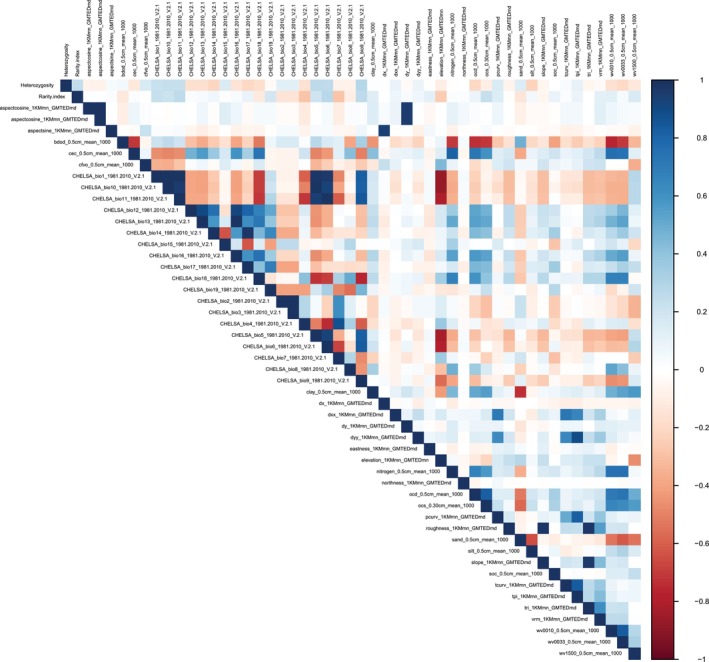
Heatmap of the Pearson correlation matrix representing the relationships between genetic diversity (heterozygosity and rarity) and environmental variables. The heatmap displays correlation coefficients, with stronger positive correlations in darker shades of blue and stronger negative correlations in darker shades of red.

The heterozygosity and genetic rarity values of the study populations ranged from 0 to 0.4 (mean 0.08) and from 0.04 to 0.32 (mean 0.09), respectively (Appendix S3). These genetic indices showed very low correlations with environmental variables (∣*r*∣ < 0.3, Figure 2). The heterozygosity ~ multivariate environment LASSO model with the optimal lambda (0.00047) included 38 variables (environmental factors) that explained 37.82% of the variance in heterozygosity values across the study populations. The model coefficients for these environmental factors were notably low in most instances, with values typically below 0.05. Exceptions to this were observed for two topography descriptors, the profile and tangential curvatures pcurv (22.18) and tcurv (−4.01) (see description in Figure 1c).

The rarity ~ multivariate environment LASSO model with the optimal lambda (0.002186) included 23 variables (environmental factors) that explained 19.59% of the variance in genetic rarity values in the study populations. The model coefficients for these environmental factors were also very low, with values typically falling below 0.05, except for pcurv (7.82) and vector ruggedness (vrm) (Sappington et al. 2007) (0.36).

**FIGURE 3:**
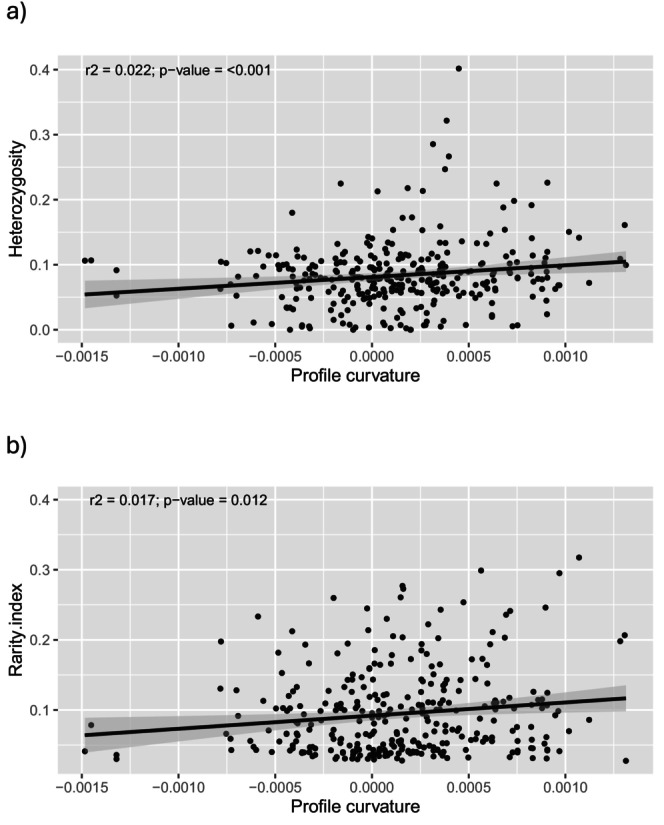
Scatterplots and linear models of the best variable from LASSO models (profile curvature, pcurv) regressed against heterozygosity (a) and genetic rarity (b) values from all populations pooled across species.

Univariate linear models, heterozygosity ~ pcurv (Figure 3a) and genetic rarity ~ pcurv (Figure 3b), were significant at *p*‐value < 0.05 (*p*‐value < 0.001 and *p*‐value = 0.012, respectively). On the contrary, they accounted for a small percentage of the variance (*r* ^2^ = 0.022 and *r* ^2^ = 0.017, respectively.

**FIGURE 4:**
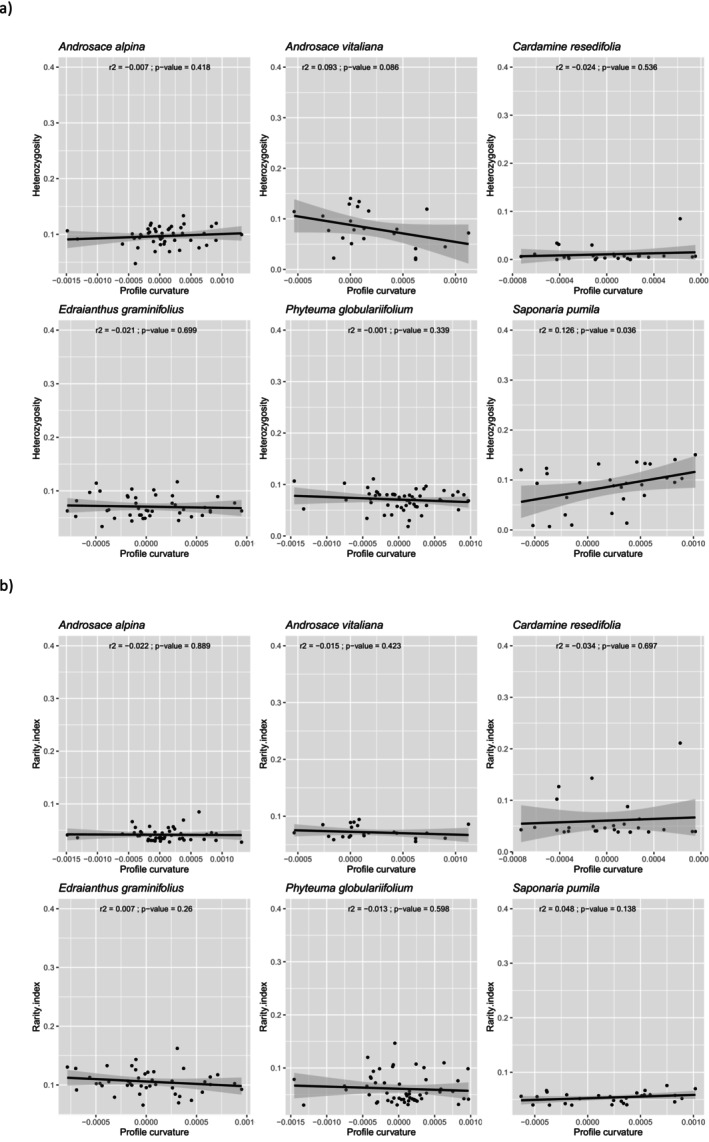
Scatterplots and linear models of profile curvature (pcurv) regressed against heterozygosity (a) and genetic rarity (b) values of six study species independently (those with > 20 populations sampled).

Among the six species with more than 20 populations, we only observed a significant positive association between heterozygosity and pcurv (at *p*‐value < 0.05) for *Saponaria pumila* (*r* ^2^ = 0.126) and a marginally significant (*p*‐value = 0.086) association for *Androsace vitaliana* (*r* ^2^ = 0.086). None of the analyzed species exhibited a significant association between genetic rarity and pcurv (at *p*‐value < 0.05) (Figure 4).

**FIGURE 5:**
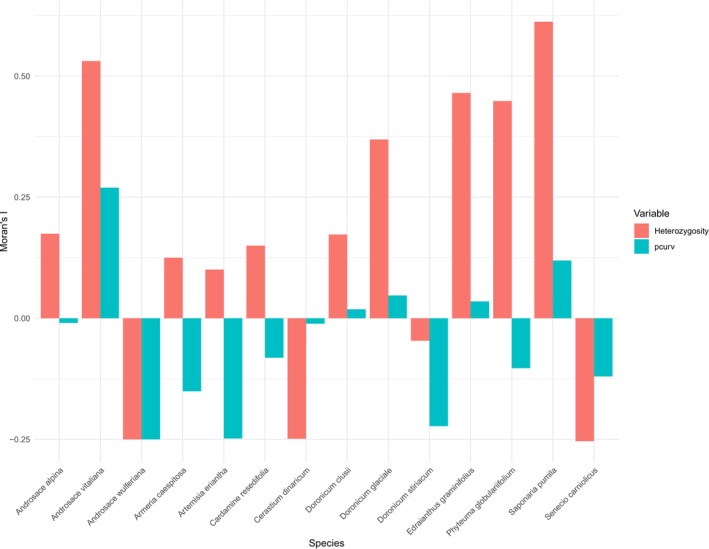
Barplot of Moran's I for heterozygosity and profile curvature (pcurv) across species. The plot shows Moran's I values, which measure spatial autocorrelation, for each species in the dataset. Positive values indicate clustering and negative values indicate dispersion.

Moran's I index for heterozygosity was particularly high in *S. pumila, A. vitaliana, Edraianthus graminifolius*, and *Phyteuma globularifolium*, in that order. This index was also high for pcurv in *A. vitaliana* and *S. pumila* (Figure 5).

## 4 Discussion

In this study, we aimed to uncover relationships between genetic diversity and environmental variables across European alpine plant species. Our findings indicate that genetic diversity (heterozygosity and rarity) is only marginally associated with one measured environmental factor. The best predictor variable selected by the LASSO models was pcurv. This variable determines the concavity (areas susceptible to deposition) and convexity (areas susceptible to erosion) of topography at the population sites. It is strongly correlated with topographic position index (tpi, Figure 2), which compares the elevation of a cell to the mean elevation of the sorrounding cells, a common variable representing erosion and deposition. We observed significant associations when examining the relationships between this variable and the genetic diversity parameters across all populations from all species combined. However, at the species level, we only detected a significant association with heterozygosity in the case of one species, *S. pumila* (Caryophyllaceae).

High spatial autocorrelation can lead to spurious associations between genetic indices and environmental variables when both genetic values and environmental factors exhibit a shared spatial structure independently. Measuring spatial autocorrelation in this case is important to avoid misleading associations between genetic indices and the environment, as it can lead to inflated estimates and Type I error rates (Sokal et al. 1998; Rousset and Ferdy 2014; Clappe et al. 2018). This seems to be the case for the significant and marginally significant positive associations found in *S. pumila* and *A. vitaliana*.

AFLPs are dominant markers, which do not allow for differentiation between homozygous and heterozygous individuals, potentially leading to inaccurate estimates of heterozygosity. This lack of codominance makes AFLPs less ideal for studying genetic variation when compared with codominant markers such as microsatellites or SNPs, which offer more detailed insights into allele frequencies. Another limitation concerns the relatively small sample sizes, ranging from 20 to 56 populations in association analyses. While this number may be sufficient for some studies, it may reduce statistical power when attempting to detect genetic‐environmental associations at the species level. Additionally, small sample sizes within populations can limit the ability to capture the full genetic variability of a population, increasing the risk of missing subtle but significant associations. It is also possible that our analyses may have missed key variables that could have revealed significant associations. In addition, the effectiveness of this method might differ in various environmental settings and geographical scales. It is important to note that a previous study indeed demonstrated a global‐scale association between genetic diversity and global biomes (Miraldo et al. 2016).

We showed that associations between genetic diversity/rarity and pcurv were not generalized across species, but we identified a significant association when populations from all species were pooled together. While we suspect that the significant association found could be spurious, the finding of a general pattern of higher genetic diversity in more convex topographies (susceptible to erosion) than in concave topographies (susceptible to deposition) could be explained by several reasons. First, areas susceptible to erosion are usually mountain peaks that can act as geographic barriers restricting gene flow among populations. Populations colonizing high mountain peaks may start from a small number of individuals (founder population). This limited genetic pool can result in higher genetic rarity through increased genetic drift compared with populations in concave locations (usually valleys) with larger population sizes. Also, topographies susceptible to erosion are areas with challenging environmental conditions that could impose unique selection pressures. Over time, these selective pressures can favor rare but advantageous genetic variants under these conditions while maintaining heterozygosity for other loci (Byars et al. 2007; Gonzalo‐Turpin and Hazard 2009). At the same time, if these founders come from different source populations, they may bring a diverse set of alleles leading to high heterozygosity within the population (Thiel‐Egenter et al. 2009).

Although we used an extensive dataset of environmental predictors, we could not pinpoint clear environmental variables suitable for use as proxies for the genetic diversity and genetic rarity of the species analyzed at the species level. Consequently, using such variables for decision‐making in conservation efforts in European alpine areas, including on‐site population protection or population sampling for long‐term storage in seed banks, is not recommended. We used heterozygosity and rarity from AFLP markers under the assumption of associations between these indexes and the adaptability of the populations. Other studies in alpine plants have identified adaptive loci through associations between environmental variables and particular AFLP allele frequencies (Manel et al. 2012). It is interesting to note the significance of associations with AFLP alleles but not with population genetic diversity indexes. This could indicate that fixation of certain alleles drives adaptation, and therefore genetic diversity indexes are not good proxies of environmental adaptability. These indexes could only reflect the effect of complex demographic histories in areas affected by glaciations (Taberlet et al. 2012).

Recently, it has been argued that in most cases census size data can be applied to obtain a valid proxy of genetic diversity (Hoban et al. 2020, 2023). It has also been remarked that proxies are not sufficient but complementary when genetic data is not readily available. There are hardly any shortcuts to identifying accurate patterns of genetic diversity in high‐mountain ecosystems. Detailed assessments of selectively neutral and adaptive genetic markers for numerous species would be necessary to inform conservation policies adequately and to try to meet the proposed goal of the United Nations of protecting 90% of genetic diversity for all species by 2030.

## Author Contributions

**J. L. Blanco‐Pastor:** conceptualization (lead), data curation (equal), formal analysis (lead), investigation (equal), methodology (lead), supervision (lead), writing – original draft (lead), writing – review and editing (lead). **J. Fajardo:** formal analysis (equal), writing – review and editing (equal). **A. G. Fernández de Castro:** formal analysis (equal), writing – review and editing (equal). **M. Fernández‐Mazuecos:** data curation (equal), writing – review and editing (equal). **J. M. García‐Martín:** data curation (equal), writing – review and editing (equal). **I. M. Liberal:** data curation (equal), writing – review and editing (equal). **A. Otero:** data curation (equal), writing – review and editing (equal). **J. V. Sandoval‐Sierra:** data curation (equal), writing – review and editing (equal). **I. Villa‐Machío:** data curation (equal), writing – review and editing (equal). **J. C. Zamora:** data curation (equal), writing – review and editing (equal). **S. Martín‐Bravo:** data curation (equal), supervision (equal), writing – review and editing (equal).

## Conflicts of Interest

The authors declare no conflicts of interest.

## Supporting information


**Appendix S1.**—Summary of genetic data used in the study, including the number of populations sampled and the number of loci analyzed for each alpine plant species. References to original data sources are provided, including DOIs for accessibility.

**Appendix S2.**—Raw AFLP (Amplified Fragment Length Polymorphism) genotype data for individual plants across alpine species used in the study. Each sheet corresponds to a different species, listing individuals, population identifiers, and binary allele presence‐absence data across loci.

**Appendix S3.**—Population‐level summary statistics and environmental variables for the study species. This table includes measures of genetic diversity (e.g., heterozygosity, rarity index) alongside geographic coordinates and topographic and soil‐related variables.

## Data Availability

Data to replicate the study is available as Supporting Information (see Appendices S1–, S3).
